# A Qualitative Exploration of Refugee Experiences of Access to a Dedicated Multi-Disciplinary Refugee Health Team in an Australian Context: Implication for Future Care

**DOI:** 10.1177/2752535X241286250

**Published:** 2024-09-19

**Authors:** Jennifer White, Ashley Young, Murray Webber, Joy Harrison, Amy Hiscox, Jessica Lush, Baeho Joo, Janessa Sherrin, Mattias Grasselli, Julie Byles

**Affiliations:** 1 College of Health Medicine and Wellbeing, 5982 University of Newcastle, Callaghan, NSW, Australia; 2 Hunter Medical Research Institute, Newcastle, NSW, Australia; 3 Multicultural and Refugee Health Service, 5260 Hunter New England Local Health District, Wallsend, NSW, Australia; 4 Refugee Health Program, John Hunter Children’s Hospital, 5260 Hunter New England Local Health District, New Lambton, NSW, Australia; 5 Refugee Health, 94516 Armidale Community Health Centre, Armidale, NSW, Australia

**Keywords:** access to care, allied health, cultural competence, interpreters, qualitative, refugee

## Abstract

**Aims:**

Refugees experience physical and mental health issues that need attention following settlement in a new community. However, access to and utilisation of healthcare services is challenging. We aimed to explore the experience of refugee access to a dedicated multi-disciplinary refugee health team.

**Methods:**

An interpretative qualitative study. 17 qualitative interviews were conducted with Ezidi refugees who attended a newly established multi-disciplinary refugee health program in a regional town in NSW, Australia. Data were analysed using an inductive thematic approach.

**Results:**

Participants (*n* = 17) identified as Ezidi and were from Iraq. Parents were between 23 and 57 years of age and had 1–12 children per family. Most had been in Australia between 2 and 5 years. Four key themes were identified: (1) Identifying the extent of health needs following a long wait to migrate; (2) Health support across the life span: the benefit of access to a multi-disciplinary team; (3) Gaps in cultural competence – impacted by understanding and interpreter access; and (4) Ongoing health and lifestyle concerns – influenced by understanding and education.

**Conclusions:**

We identified the benefit of access to allied health for prompt diagnosis, treatment and management of conditions including congenital and developmental conditions, mental health and chronic diseases. Access to a dedicated team ensured early intervention for a broad range of health and social issues including early referral to services, close coordination and help to complete supporting paperwork and applications. Ongoing investments are needed to maintain this comprehensive and coordinated approach to care that is underpinned by a family centric approach.

## Introduction

Even in the safety of resettlement in a new country, such as Australia, the ongoing health and wellbeing of refugees is closely associated with their ability to access high quality, coordinated health care.^1^ Previous evidence demonstrates that newly arrived refugees struggle to access health care including general practitioners (GPs), specialists, community health services and hospitals due to language barriers, cultural differences, and the complexity of understanding the Australian healthcare system.^2–4^ Further evidence shows that the provision of quality primary care is inconsistent and that many providers have limited knowledge of refugee health requirements^5^ which is compounded by increasing demand.^6^ Additional challenges include inadequate access to interpreters,^7^ and delayed or fragmented care coordination between sectors.^5^

The delivery of culturally competent care is a challenge to many countries experiencing increasing numbers of migration, including refugees who have been forced to leave their home country. Australia has a diverse and growing migrant population with 190 different countries and 300 different ancestries represented.^8^ Clinical concern stems from evidence demonstrating that health care access among people from culturally and linguistically diverse backgrounds (CALD) with low English proficiency (LEP) is characterised by longer hospital stays, increased diagnostic testing, higher rates of medical errors, and poorer patient satisfaction.^9–12^ The provision of a culturally competent health care service has been posited as a key factor towards improving health outcomes in CALD populations, increasing the efficiency of clinical staff, and improving patient satisfaction. Key organisational components shown to promote cultural competency include the provision of health professional training and creating policies that streamline care and facilitate communication.^13^ However this can be difficult in the context of providing care to refugees with challenging health care needs.^14^ Barriers towards the provision of culturally competent care include a lack of value in and resources towards staff training and education alongside the inadequate provision of culturally appropriate health education materials and other supports to help CALD patients, including refugees, navigate the health system.^14,15^

Australia is a major site for the resettlement of refugees. As part of Australia’s humanitarian resettlement program, 4, 123 Ezidi people have been given offshore Humanitarian visas since July 2021.^16^ Building on existing challenges towards the provision of culturally competent care is the need to meet the specific needs of refugee populations and help them access an unfamiliar health care system. The Australian government’s decision to resettle refugees and migrants in rural and regional areas has been debated in part due to the capacity of the health system to respond. Indeed, Australians living in regional and rural areas have been shown to experience poorer health status compared with metropolitan residents, compounded by inequitable access to primary health care services (PHC).^17,18^ Effective access to health care is critical for refugees, such as the Ezidi people who fled their home following the horrific attacks by the Islamic State of Iraq and Syria (ISIS). The Ezidi people experience ongoing complex, health issues as a result of their experience of persecution, trauma, living in unhealthy environmental conditions and disrupted or inadequate access to health care.^19–21^ Health issues may include, but are not limited to, mental health conditions, the impact of sexual violence, injuries, malnutrition, and the ramifications of poorly managed existing health conditions and noncommunicable diseases.^22^ Access to health care in a foreign country is difficult and recent evidence highlights potential barriers in accessing and engaging with healthcare services due to lack of language skills, poor access to health information, misconceptions about diseases, unemployment and lack of autonomy.^2,14^ Additional evidence highlights the extent to which the ongoing experience of poor physical and psychosocial health impacts on the capacity of refugees to effectively adjust and transition to a new country and obtain accommodation, learn a new language, study and gain employment.^23,24^ Some specialised services have been established in Australia to help refugees access healthcare however the majority of healthcare for refugees occurs within mainstream services located in large metropolitan areas.^25^ Even despite the availability of health care services in regional settings, albeit limited,^25^ research demonstrates that refugees underutilise health services due to a lack of familiarity with the health system and reduced ability to self-advocate.^14,26^ This is compounded by poor health literacy, knowledge about community health resources, costs and access to transport.^26^

While there remains a shortage of adequate comprehensive services for refugees, new initiatives have been developed in many Australian regions. Such initiatives are often as a pragmatic response to the needs of both refugees and health providers.^27^ While all refugee resettlement sites in Australia provide an initial assessment by a nurse within 28 days of arrival, in 2019 the Hunter New England Local Health District obtained funding to establish a part-time occupational therapist (OT), physiotherapist (PT) speech pathologist (SP) and social worker (SW) to help meet the needs of the Ezidi refugee population in Armidale, NSW. There is limited research examining the experiences of Australian refugees.^14^ Also to our knowledge there are no other settlement sites in Australia that provide dedicated allied health services. In response, this paper aims to explore refugee experiences of access to the Armidale Refugee Health Program, including potential benefits, and gaps in service delivery.

## Methods

This interpretative qualitative study^28^ employed the use of semi-structured interviews and was informed by the Consolidated criteria for reporting qualitative research (COREQ) checklist.^29^ This study used a constructivist paradigm to understand Ezidi refugee experiences of access to the Armidale Refugee Health Program, including potential benefits, and gaps in service delivery.^30,31^ We chose an interpretative, constructivist approach which best explores how individuals or groups construct reality based on interactions with the social environment.^28^ By exploring the social reality of the Ezidi refugees we were able to explore how they made sense of their experiences in order to reveal new understandings.^32,33^

For pragmatic reasons we used a purposive sampling to strategy to identify Ezidi refugees of varying ages with differing health experiences to capture a diverse range of perspectives. Careful attention was paid to ethical considerations given the sensitivities of conducting research with refugees. As a result, we used a multistage informed consent process. Members of Armidale Refugee Health Program, who had established relationships with the local Ezidi population based on trust, phoned potential participants who had utilised the Program themselves or had children who had been seen by the team. Phone calls were made with the assistance of a NAATI (The National Accreditation Authority for Translators and Interpreters Ltd) trained interpreter to explain the study, ethical considerations and invite participation. Participants who provided verbal consent were later phoned to confirm the interview and re-confirm consent. Written informed consent was obtained prior to the interview including consent for interviews to be audio-recorded. Approval for this project was obtained from the Hunter New England Health Human Research Ethics Committee (2021/STE02669).

### Data Collection

Interviews were conducted at the Armidale Community Health Centre by an experienced qualitative researcher (JW) For ethical reasons participants were given the option of having a trusted member of the Armidale Refugee Health Program, familiar to the participant, sitting in. However no participants took up this option. All interviews took place in a private room to prevent intrusion or distraction and to ensure participant privacy, comfort and anonymity. To minimise the risk of distress, interview questions did not initiate discussion of traumatic experiences. After asking demographic questions to help establish rapport, the interview progressed. Open-ended question, using an Interview Guide (see Table 1) explored how the family perceived their health challenges, what services they had accessed and why, and their hopes for the future with regards to health service access. Although ethics approval included scope to interview children, for pragmatic reason only parents were interviewed, thus representing their own health needs and that of their families. This decision was made following pilot interviews where it was observed that participants were uncertain of how health care services were accessed, including a lack of understanding regarding the nature of the professional groups who provided services. We felt this uncertainty would be amplified in children. In response, we triangulated participant reports of services accessed with the verification by the Armidale Refugee Health Program of the services provided.

**Table 1.** Interview Guide.

Question	Prompt
Can you tell me a bit about your health condition?	Can you tell us about your health condition and how it impacts you?
	Does it get in your way? Impact on work, school etc
	Describe e.g. pain, disability etc
	If you have any health problems, who is the first point of contact for you and why?
Can you tell me a bit about your understanding of why you were referred to [name discipline] of Armidale refugee health program?	Can you tell us about the people who helped look after you?
	What was the reason for your referral to Armidale refugee health program?
	Did you understand why you were being referred?
	How do you feel about this? Expand
	E.g. worried, anxious, confused?
Can you tell me how you managed your health before you saw the Armidale refugee health program?	Expand
Can you tell us about experience with the Armidale refugee health program?	Were they able to help you?
	What helped or did not help?
	Do you have ideas how they can help you more?
Do you have any other health needs that you feel are not being addressed?	Please describe?
	What would help you?
If you could change one thing about the healthcare services you received, what would it be?	
Do you have any further comments	

### Data Analysis

Interviews were audio-recorded, transcribed verbatim, and checked for transcription errors. Data was coded using an inductive thematic approach by two authors (JW, JB). Analysis followed a three-phase approach involving (i) identifying units of meaning by reading the transcripts line-by-line, (ii) grouping units into codes to assist with data retrieval, irrespective of the research question, and (iii) examining relationships between codes to themes. Using a word document, codes was issued with a four-letter label or code to facilitate data retrieval between the transcripts (e.g., the experience of gratefulness was given the code GRTE). Categories were identified following the exploration of connections between the codes (e.g. all codes that identified uncertainty with the health system). Through regular discussion with broader team, the final step examined relationships between categories to form themes. Coders captured exemplar quotes supporting each theme.

### Rigor

Trustworthiness of our data was facilitated using several strategies, including immersion in data; reflexive analysis, and peer debriefing.^34,35^ The team met regularly to practice reflexivity and discuss any issues with bias during data collection, analysis and interpretation. Team discussions informed saturation, and recruitment continued until no new categories arose from interviews. Credibility and confirmability were attained by transparent and precise record-keeping, including detailed field notes and a personal reflective journal of the researcher (to ensure that no context was lost when analysing interview data). The study’s transferability was reinforced by careful sample selection to ensure the participants were as diverse as possible. Member checking was not undertaken to avoid feelings of distress that may arise when reflecting on experiences.

## Results

In total, 17 semi-structured interviews were conducted. All participants were recipients of humanitarian visas, having been sponsored and supported by the Settlement Services International (SSI) for refugees. Limited demographic characteristics are outlined to ensure anonymity. All participants identified as Ezidi and were from Iraq. Parents were between 23 and 57 years of age and had between 1–12 children per family. Most participants had been in Australia between 2 and 5 years. Parent education levels ranged between year 6 and 12 school levels in Iraq. All participants were on some form of welfare, with limited opportunities for unskilled work such fruit picking or labouring.

Four key themes were identified related to refugees’ access to the Armidale Refugee Health Team and the broader health system.

1. Identifying the extent of health needs following a long wait to migrate
2. Health support across the life span – the benefit of access to a multi-disciplinary team
3. Gaps in cultural competence – impacted by understanding and interpreter access
4. Ongoing health and lifestyle concerns – influenced by understanding and education

We have used data extracts from the interviews to illustrate each theme. For ease of reading, the English has been corrected from the original spoken form, which had many grammatical errors, and had been translated into English from the participants’ own language.

### Identifying the extent of health needs following a long wait to migrate

#### Experience of a New Health System

All participants reported feelings of “stress” (Participant (P) 11) towards how they would access health care in a new country. Difficulties were attributed to lack of familiarity with the health care system, language barriers and feelings of isolation from previously known support systems. Closely aligned was concern towards adequately accessing health care needs for family members.

It was very difficult; we didn’t know the language and it was the first time for us travelling in our life. It was a new country, and we didn’t have anyone to support us. It was a very difficult time. (P8)

I keep thinking about the kids, their pain and the family’s problems. (P1)

Despite the uncertainty attributed to unfamiliar processes towards health care access, participants were reportedly confident they would get the help they needed. Feelings of confidence were based on what they had heard or been taught in their home country or “refugee camps”. (P13)

We knew that this [Australia] is a very good country, and everybody has the right to get treated with respect and dignity. We knew they would help us here and give us the support we needed. We are very thankful for everything done for us. (P14)

Most participants reported experiencing a range of barriers towards accessing health care in their country of origin, Iraq and were hopeful about the opportunities for access to health care on arrival in Australia. Such barriers included perceived limitations regarding accessing doctors, in part due to distance, cost and availability of medical technology.

The equipment (technology) is more advanced here (Australia), there is access to more, kind of, up-to-date equipment. Also, the doctors here have more experience. The doctors back in Iraq, they have to advance their English before they can learn how the (new) equipment works. (P1)

No, back home in Iraq we would hardly see doctors often. If we were in pain, we just moved on because it was too much effort to go to the doctor. (P2)

Many participants reported they waited until they arrived in Australia in order to access treatment that had been difficult obtain in Iraq especially due to cost and difficulty accessing specialised services. Examples included the need for orthopaedic surgery, post-polio treatment, cochlear implants and heart surgery. Participants expressed gratefulness for being able to receive treatment that enabled them to embrace life in a new country, giving them confidence to be able to engage in school and work.

In Iraq they wanted to do a surgery, but we had to pay for it, and we couldn’t afford paying for the surgery. So, we waited until we came to Australia, and we received the surgery here. We are very thankful for all the support. (P11)

#### Early Support Needs

Most participants reflected on the support they received from SSI and the Armidale Refugee Health Nurse assessors and SW who were their first point of contact with the Australian Health system. Key benefits reportedly included receiving an initial assessment of health needs for all family members, having referrals coordinated and gaining access to a local General Practitioner (GP). Indeed, the Armidale Refugee Health Program played a critical role in negotiating with and advocating for local GPs to accept refugees as new patients, even when their books were closed. Paperwork to register with a GP and apply for their medical history from Australian Affairs was completed by the Armidale Refugee Health Program which reportedly promoted timely access. As such all refugees in Armidale gain access to a GP in contrast to other Australian settlement cities where access to a GP is initially through a refugee health clinic and then refugees are required to access GPs on their own accord.

When first we came here, [the nurse] was supporting us. She helped us with all the paperwork and health care records. She arranged the appointments for us, and she wanted to make sure that we got all the support that we needed …to find the causes of the problems we are having and make sure we got the right treatment. (P12)

In the first instance, participants reported that access to the Armidale Refugee Health Program nurses and SWs was critical for help accessing GPs especially with regards to help with transport. Initially, participants reported they did not have their own car, and were unfamiliar with how to access local transport. Using public transport was reportedly more challenging when required to travel to access tertiary health services in a metropolitan city. However, most participants reportedly obtained a license and a car at a later date, which promoted their independence.

It would have been difficult if we had to do it by ourselves, but they were arranging the support for us and how to get there. (P12)

The first year it was very difficult for me, I did not have a car and I was taking care of six kids. Sometimes when they had an appointment, I needed to walk to the school to get them from school and then walk to the appointment. Sometimes it was raining, and the weather was very cold, it was just very difficult to walk through that weather. I think that was the hardest thing. Since I got my car, and my driving license, things have got better. (P15)

Most participants reported that they were inundated with the need to attend appointments for themselves and their family on arrival. However these were reportedly closely coordinated by the Armidale Refugee Health Program.

Yes, it was [the nurse] who found a GP for us and then the GP referred us to the specialist. [The nurse] helped arrange our appointments and made sure we are seeing the right person. In one month, I had seven appointments and [the nurse] organised them all. (P12)

Once established with a GP most participants were reportedly able to independently seek assistance for health care assistance for themselves and their family as required.

When first we came here, [the nurses] were supporting us, they were making the appointments for us and helping us with that. But now we know how to do it. So, whenever we need to make an appointment with the GP for our kids, we are able to do that and take them to the appointment, but in the beginning, they were helping us with that. (P9)

#### Comprehensive Health Assessment

For many participants the initial assessment, including blood and pathology tests, was an opportunity for review and management of existing health concerns. Furthermore, the initial assessment provided participants with an opportunity for health concerns to be identified. For example, some participants were diagnosed with heart disease, diabetes, intellectual disability or vitamin deficiencies for the first time on arrival in Australia. Most participants readily embraced the treatment they required in order to recover and progress their life in Australia.

I have some issues with vitamins [deficiency]. When I arrived, I couldn’t move properly because I had some issues with my back. The [vitamin deficiency] make the bones ache and become weak. (P13)

So, the main thing would be my leg. I wanted to get my leg fixed….and be independent. (P2)

In fact for some participants, access to the Australian health care system enabled diagnosis and management of complex health issues such as epilepsy, asthma, stroke post birth, deafness, cerebral palsy and chronic incontinence. In many cases participants reported that such conditions had been over-looked, under-treated or not diagnosed in Iraq.

Yeah, so, they didn’t diagnose it [epilepsy] there [in Iraq]. [My daughter] would get headaches and would fall over with - but we didn’t know the reason. I would have taken her to the doctors, and they would just give her flu medicines, something like Panadol. I would also put a cold wash on her forehead. (P5)

We were told [once in Australia)] there was spot of blood somewhere on her brain that wasn’t moving [occluded] …. that caused her [daughter] right side to feel weak…. back in Iraq they didn’t tell us much. They just told us that her brain is not developing well. (P9)

While participants were grateful for the support they received on arrival, they also reported feelings of distress and confusion. Such feelings were heightened in the early stages of their settlement, when participants reported being overwhelmed and disorientated. Participant reports highlighted the challenge of accessing health services, even when transport had been arranged, when unable speak English. Indeed some participants wondered if the stress of arriving to a new country caused their health problems.

We didn’t know the language and once I had an appointment, the SSI arranged a driver to take me to the appointments. I thought the driver would stay with me until I finished the appointment, but he just drove me and then left. It was very hard; I didn’t know what I should do. (P14)

I missed my train and there wasn’t anyone who would support me or help me. It was a really difficult time for me. (P8)

When first we came here also, we had a health check-up and there weren’t any problems. But it was a very stressful time, and we were always anxious, so maybe that caused the problems that we were having. (P12)

#### Benefit of Access to an Existing Ezidi Community

Many participants reported that the support from the local Ezidi community was instrumental in facilitating their transition and familiarity with the how to access local health care and “give us some advice” (P13). Participants noted the benefit of knowing existing members of the community who were often able to speak some English and could explain the heath care process, provide support by attending appointments, or had a car to help with transportation.

So, initially it was other people who came here before us, it is the community people who arrived here before us, they were very helpful and explaining things and telling us this is what we do here. (P1)

### Health Support Across the Life Span – the Benefit of Access to a Multi-Disciplinary Team

#### Benefit of Early Access

Access to the Armidale Refugee Health Program promoted early access to allied health in contrast to being placed on a waiting list for community services including OT, PT, SP, SW and psychology. Indeed, long wait lists typically applied for allied health in community health. Participants also valued support from the Armidale Refugee Health Program to negotiate access to visiting specialists and avoid travelling long distances, to unfamiliar cities.

Yes, we were planning to go to Newcastle (metropolitan city) to see some specialists, but they arranged for us to have the appointment in Tamworth, (regional city) with a visiting specialist. (P10)

Early access facilitated assessment and treatment including access to physical therapy components that had an immediate impact on wellbeing. For example, the prescription of adaptive equipment for existing disabilities facilitated “independence” (P3) which hadn’t been possible in Iraq where such equipment was not available.

We have chair that you can sit in the shower and wash. We have never seen this stuff [equipment] in our country. The government that we grew up in, we did not get any help with anything and this….so the Australia Government has been helping us a lot. (P2)

For other participants, especially families who had children with disabilities, allied health treatment promoted gains in achieving developmental milestones such as training in activities of daily living (ADLs). Such therapies were reportedly not available in Iraq and were observed to have an immediate impact on independence. Ongoing therapy was reportedly integral as children aged for example the therapeutic instruction of one child with development disabilities to use sanitary products.

[Therapist] was showing her [daughter] how to wear her underwear and take them off and what to do. (P9)

#### Help for Complex Needs

Early access to allied health was reportedly beneficial for trauma specific care including mental health and for injuries previously sustained during conflict. Physical therapy and access to OT and PT facilitated treatment for conditions such as chronic pain and strengthening and mobility exercise addressing impairments resulting from past accidents, war trauma, or medical conditions such as polio.

So, my problem is my leg (post-polio). When I walk my heel does not touch the ground, cannot touch the ground so I have to walk on my toes. He (physio) made me a boot for that leg - so when I was walking with the leg in the boot, I was walking straight. (P2)

I had a bullet in my back, so it’s been seven years now, and since then I have been getting the chronic back pain. (P3)

Indeed, early access promoted the identification and management of specialised care needs resulting from trauma. For example one child experienced an eating disorder and was supported by allied staff to engage with early intervention underpinned by a holistic approach. This child was able to access an OT, SP and dietetics to facilitate education of her parents towards eating and obtaining a nutrient intake. Likewise, the team reportedly advocated access to childcare, including completing paperwork and applications for fee subsidies. Attending childcare was reportedly invaluable and allowed this child the opportunity to observe and mimic other children playing and eating.

So, they were playing with her…bringing her different foods and seeing which one she is going to eat, organising childcare. So, they helped us a lot. (P1)

All clinicians played a key role in facilitating the National Disability Insurance Scheme (NDIS), a scheme of the Australian Government that funds all costs associated with disability application for long term support. Access to allied health was critical for assisting with arranging assessments to help confirm a diagnosis and complete paperwork. Such processes were reportedly difficult without adequate language skills or health literacy.

She can walk, she talks, but her talking and her age do not match. She just started becoming a client with the NDIS. The refugee health team have helped us with the application for the NDIS. (P10)

In addition to acute issues participants benefitted to education toward the management of chronic disease such as heart disease and diabetes.

When we go to our GP, they will tell us … this is good and this is not good thing for your health [previously not addressed in Iraq]. (P12)

### Gaps in Cultural Competence – Impacted by Understanding and Interpreter Access

#### Understanding Team Roles

All participants expressed gratitude at being able to access a diverse range of nursing and allied health services however they reportedly continued to struggle to understand the role of each team member.

So, the health system that we have been needing has been good and we kind of understand it. The only problem is that we don’t know who everyone is, but everyone’s been helpful, everyone in the team have been very equally helpful. (P1)

#### Interpreter Challenges

While most participants reported that they always had access to an interpreter, either in person or over the phone, others reported scenarios when they did not have access to an interpreter such as when presenting to the maternity ward or the Emergency department (ED.). Over time many participants had grasped some English, facilitated by the compulsory TAFE (Technical and Further Education) English course, but still struggled when an interpreter was not available. When an interpreter was not available participants reportedly required help from family and friends.

When I gave birth, it was Saturday and they told me there was no interpreter available because it’s the weekend. So, my friend came with me. (P16)

In ED you have to wait. So, after he [son] was getting worse, I was scared. I mean I can try my best (interpreting) for me and my family but (it’s difficult) …. (P13)

Other participants reported that even when an interpreter had been booked, they often didn’t speak Kurdish Kurmanji, which meant they were not able to understand. One participant explained that men had more exposure to different dialects through their work in Iraq, in contrast to women who were often at home. As a result this participant reflected on importance of having access to an interpreter who spoke the correct dialect language when providing health care to women. This was further highlighted when participating in telehealth appointments when the benefit of communication through body language was removed.

It’s hard to explain, so we mostly use body language. If on the phone, we can’t see each other, so it’s hard. Even if the sound is different, especially for women, who haven’t worked. For men, yeah, they work with Iranian, Syrian and Turkish people, so we know their sounds, but women never have, so that’s very difficult for them. I prefer face-to-face [interpreters]. (P13)

In response, appointments had the potential to be cancelled leading to delays in health care access.

Lots of appointments we cancelled because of that [incorrect interpreter]. (P13)

#### Navigating Appointments

Many participants reported difficulty with the booking system for health care appointments. In most cases participant reported they were sent appointment information or reminders via SMS (short message service) and sometimes mail, which was challenging if they lacked proficiency in English. A phone call reminder was perceived to be most helpful.

There were a lot of times I forgot about my appointments so even though they send me like text messages like a reminder, but sometimes it is hard for me to understand. Sometimes I will give it my kids and ask them to read it for me. When they give me like a call to remind me of the appointments that is helpful, it will be easier for me to come to my appointments but when they send me text messages sometimes it is hard. (P17)

Indeed, many participants reportedly relied on their children to interpret and manage appointment bookings and attendance. Alternatively, they used “Google translate to translate something.” (P15)

Initially we were getting help with the appointments and everything, but now my daughter, she is in charge of making the appointments and taking us to the appointments. (P5)

In particular, participants who were from large families reported difficulties in interpreting and balancing multiple appointments. Such issues were exacerbated by the Australian primary care system and the Medical Benefits Scheme (government subsidised care) which only allows the item that best describes the service to be claimed. This was perceived to be challenging and added burden to participants, especially those who coordinated care for their whole family, when they desired multiple issues to be addressed in a single appointment. While participants reports didn’t label their experiences as carer burden their expressions highlight the emotional and practical burden of caring for others and facilitating access to multiple appointments. Many male participants reportedly adopted the role of carer and reported occasions when they had to leave work or TAFE to assist their family. This was an additional source of stress and concern when trying to progress learning English and earning a wage.

My husband is a full-time worker, and he is the one who drives. So, whenever she (daughter) has an appointment, he has to leave his work and drive them to the appointment. (P9)

Most of my time is spent for taking people to appointments… so I have to take time from my TAFE to take someone to the appointment, bring them back, take the other one. It’s very difficult to remember things and memorise things. If I didn’t have these issues (worry and support of family), I would do much better at TAFE. (P1)

### Ongoing Health and Lifestyle Concerns – Influenced by Understanding and Education

#### Health Literacy Challenges

The extent to which participants reportedly engaged with the health care system was experienced on a spectrum. Heath service engagement was impacted by gaps in health literacy and understanding of what services where needed and available. Some participants, especially in the first months of settlement, reported they were constantly and actively negotiating an overwhelming system to try and coordinate care and appointments for the whole family. Other participant reports suggested they were passive and not actively engaged which resulted in ongoing concerns and unanswered questions. For example, one participant reported her daughters had all experienced facial injuries from a car accident that occurred while fleeing ISIS. As her daughters aged, they became very self-conscious about their scars from the accident and the participant was uncertain how to access help for them.

I was hoping they [health care system] do a kind of cosmetic surgery for my daughters because their scars are pretty obvious. (P5)

Participants reported feelings of confusion and frustration when they didn’t understand the health system. Challenges in engaging with health professional was also compounded by LEP, which meant that some participants never asked questions. For example, several parents of children with complex needs reported they didn’t understand their child’s prognosis but didn’t ask questions. This led to misunderstandings such as understanding why therapy had ceased. Similarly, a few participants reported they were concerned about the quality of therapy when they didn’t get the result they craved.

[My daughter] was seeing [Speech Pathologist], but she didn’t improve much. It’s been a while, and we haven’t seen [Speech Pathologist]. We are not sure why; we don’t know what happened. (P8)

Yes, we have worked with a caseworker from NDIS. The case worker said they told us someone who will see [daughter] for her speech. We told them we want to see someone [Speech Pathologist] who is really who is really good that can help her (as they perceived past therapy has failed). (P10)

#### Long Term Needs

Likewise, participants struggled with access to NDIS and how to advocate for services needed. Many participants expressed concern about the delays in getting approved for NDIS and understanding what services would be available to help their children.

It’s always very difficult when you have the kids, so the sooner they provide the help for them the better. It makes their life easier, and if there is like anything else they think is good for them, it would be really good to provide for them. (P11)

Overall, many participants were concerned about their future and how to secure more consistent or permanent housing.

One day if we wanted to apply to get a new house would we be able to get more support for that? Or not? (P8)

## Discussion

This study explored the experience of refugee participation in a dedicated multi-disciplinary refugee health team. Results highlighted the benefit of early access to support and intervention towards the management of existing or emerging health conditions. Key factors included prompt assessment and treatment of identified health concerns, as well as facilitating access to ongoing key services such as GPs and the NDIS. Specifically, a key role of the multi-disciplinary team was the ‘behind the scenes work’,^36^ necessary to collect demographic and health assessment data and complete the extensive paperwork needed to access services, especially NDIS. Indeed, many Ezidi refugees were not sufficiently proficient in oral or written English, nor had they arrived with the supporting heath and identification documentation required to complete complex paperwork. Refugees continued to struggle with understanding health system process and often had unanswered questions about future needs. Husbands and children took on carer roles which were often burdensome.

Our findings echo with previous studies highlighting the extent of physical and psychosocial health needs of refugees.^37,38^ In this study refugees experienced ongoing psychological conditions, including post-traumatic stress disorder (PTSD), and physical conditions such as developmental conditions, musculoskeletal and pain issues, injuries from trauma related events, scars and malnutrition which benefited from care coordination by the Armidale Refugee Health Program. Key care coordination tasks included assessment and screening towards the extent of health and social needs, making referrals, providing treatment and ensuring follow-through of care.

The significant health needs among people from refugee backgrounds has been previously documented including difficulties accessing primary and complex care.^39^ In this study, access to a dedicated multi-disciplinary refugee health team meant that vulnerable people did not have to experience extensive waiting times for conditions that had already been left untreated for varying lengths of time. Similarly private allied health or specialist care was sometimes not offered or provided to refugees due to the additional cost of providing interpreters. As a result, access to a dedicated multi-disciplinary team prevented people from falling through the gaps and struggling on their own. Likewise, the role and expertise demonstrated by the Armidale Refugee Health Program, is consistent with evidence towards the growing need for and availability of primary care models targeting high-needs, high-cost (HNHC) populations - where care provision is time and resource intensive and requires complex coordination.^40^ Closely aligned is the provision of care to refugees that is family centred care (FCC) where the family is the unit of care rather than individual.^41^ FCC is underpinned by shared decision making and effective communication, which can make inroads in overcoming language barriers and ensuing culturally competency. However primary care reform strategies are needed to encourage FCC such as facilitating clinicians to address multiple family members in the one appointment.^42^

Consistent with emerging evidence we demonstrated that refugees have difficulty accessing primary care and the NDIS system, systems that requires competency in English language skills, and sophisticated advocacy skills.^43,44^ The Armidale Refugee Health Program played a critical role in arranging assessment and completing essential paperwork. Indeed, it has been identified that up to 50 h of additional support is required to help refugee families meet the requirements to complete the online NDIS application process. Challenges were magnified when required to access supporting documentation towards diagnosis, medical histories of diagnosis and services that had never been obtained or had been lost or destroyed in conflict.^44^ Furthermore, while a NDIS application was critical to access help, there were also gaps in the availability of support in Armidale a regional setting. In particular there were gaps in mental health support, and this is common across settlement sites.^45^ Overall a comprehensive and coordinated approach is vital and should include collaboration between mental health agencies with social, community, and general health services.^45^

While many participants in this study readily adapted to a new health system, others appeared to take a passive role during their health consult, and this was compounded by poor health literacy and lack of awareness of available services. Such results highlight suggest the need to better support refugee participation in self-management of their health care.^45–47^ In the interim, access to a multi-disciplinary refugee team promoted seamless, integrated care that spanned the continuum of care.^48^ Similarly, results from a Canadian study showed that access to a dedicated health clinic for government assisted refugees reduced wait times to see a health care provider by 30%.^48^ However, in response to demand, referrals to non-physician specialist health care providers nearly doubled^48^ reflecting the extent of need. Consistent with results from a scoping of models addressing health equity for immigrants^19^ we identified the benefit of teamwork and interdisciplinary collaboration and the essential role in helping refugees access help for a broad range of health and social needs. The ability to provide home visiting may have facilitated the promotion of trust and rapport that underpinned future engagement by refugees with future health care and should be considered in other HNHC models.

This study generates important in-depth insight into refugee access to health care in an Australian context, especially the need for access to a community based multi-disciplinary team. Use of interpreters in this study enabled participants with limited or no English language ability to give their opinions and allow their experiences to be shared, therefore adding to the body of knowledge available. We acknowledge that refugees who didn’t participate may have had differing experiences with health care access. Likewise, the severe nature of the some of the issues discussed might not be relevant to other refugees. However, there was no indication that participants felt obliged to participate and all participants appeared willing to share their story in the interview; demonstrated by smiling, not rushing to finish the interview, and being forthcoming with sharing their experiences.

## Conclusions and Recommendations

Key findings highlight the benefit of early access to a multi-disciplinary refugee health service in facilitating early identification of physical and psychosocial health issues, making referrals, completing NDIS applications, and providing therapy. Early intervention has the potential to mitigate associated complications and promote adjustment to a new country. However, the experience of poor health literacy means that refugees remain vulnerable especially in circumstances when there is poor integration of care. Further, we posit that investments in providing health promotion and adjustment support are also warranted for refuges to work towards a positive future.

## Data Availability

Available on request.
